# Plasma Disposition of Conventional and Long-Acting Moxifloxacin in Sheep after Intravenous Administration

**DOI:** 10.5402/2012/951306

**Published:** 2012-08-29

**Authors:** C. M. Modi, S. K. Mody, F. D. Modi, H. B. Patel

**Affiliations:** Department of Pharmacology and Toxicology, College of Veterinary Science and Animal Husbandry, Sardarkrushinagar Dantiwada Agricultural University, Sardarkrushinagar, North Gujarat, Dantiwada 385 506, India

## Abstract

This study describes disposition of long-acting moxifloxacin and conventional formulations of moxifloxacin in sheep after intravenous administration in five male sheep. Long acting moxifloxacin solution (10% moxifloxacin in solution with L-arginine, N-butyl alcohol, and benzyl alcohol) and conventional moxifloxacin (10%) were injected in jugular vein. Blood samples were collected from contralateral jugular vein in test tubes containing 30–50 IU heparin (anticoagulant) periodically from 0.083 to 72 h of drug administration. Drug concentrations in plasma were determined using High-Performance Liquid Chromatography (HPLC) with fluorescence detector. The mobile phase consisted of a mixture of buffer (10 gm of tetrabutyl ammonium hydrogen sulphate per liter-deionised water) and acetonitrile (80 : 20). The buffer was 0.067M of disodium hydrogen phosphate with pH of 7.5. The flow rate was 1 mL*·*min^−1^ at ambient temperature. The effluent was monitored at 296 nm excitation and 504 nm emissions wavelength. HPLC with fluorescence detector method for plasma moxifloxacin assay was standardized with specific modification for plasma of sheep in the present study. After single-dose intravenous administration of long acting moxifloxacin the plasma concentration of 0.016 ± 0.001 **μ**g*·*mL^−1^ was maintained for up to 72 h. Conventional formulation of moxifloxacin remained in body for up to 24 h of drug administration with the level of 0.015 ± 0.005 **μ**g*·*mL^−1^.

## 1. Introduction

Moxifloxacin is a new 8-methoxy-quinolone with a broad spectrum of antibacterial activity against organisms Gram-positive and Gram-negative bacteria, anaerobes, and atypical organism such as *Mycoplasma* and *Chlamydia* spp. It has some characteristics such as a wide spectrum of bactericidal activity, a large volume of distribution, low plasma protein binding, and relatively low Minimal Inhibitory Concentrations (MICs) against susceptible target microorganisms [1, 2], The MIC of moxifloxacin for *Mycobacterium ulcerans* ranged from 0.015–0.5 *μ*g·mL^−1^ [3] and for *S. aureus* from 0.03–0.12 *μ*g·mL^−1^ [4]. It has the highest potency in its class against *Staphylococcus aureus* and *Staphylococcus epidermidis* [5]. All these characteristics have made the moxifloxacin a drug of choice to treat many infectious diseases which could not be treated with older fluoroquinolones and other antimicrobials. The disposition study gives concentration versus time profile of drug which is prime step to generate pharmacokinetics data and subsequently to establish dosage regimens. The comparative disposition of long acting and conventional moxifloxacin helps to evaluate the difference in dosage regimens of both types of drugs and their pharmacokinetic profiles in animal body. No such kind of study has been reported to address such a comparative issue in live stock species; hence, the present study was conducted to investigate the comparative profile of conventional and long acting moxifloxacin in sheep after single intravenous (IV) administration.

## 2. Materials and Methods

### 2.1. Experimental Animals

Five healthy adult male sheep weighing 38–45 kg were used in the present study. Sheep were housed at Livestock Research Station, Sardarkrushinagar Dantiwada Agricultural University, Sardarkrushinagar. The sheep were housed in well-ventilated appropriately spaced animal shed and fed with good quality fodder and concentration. Animals had free access to clean and potable water during course of experiment. All animals were acclimatized for the period of 15 days and observed clinically daily to confirm any illness or disease. Animals were not treated earlier by any drugs. The experimental protocol was approved by IAEC and all the measures for the well being of experiment animal were taken as per CPCSEA guideline. 

### 2.2. Drug and Chemicals

Long acting moxifloxacin (10% moxifloxacin in solution with L-arginine, N-butyl alcohol, and benzyl alcohol) and conventional moxifloxacin injectable solution, moxifloxacin-base powder for standardization, were obtained from INTAS Animal Health, Gujarat, India. Water, acetonitrile, and tetrabutyl ammonium hydrogen sulfate of HPLC grade were purchased from S. D. Fine Chem. Ltd, Mumbai. 0.067 M disodium hydrogen phosphate and hydrochloric acid of analytical grade were purchased from S. D. Fine Chem. Ltd, Mumbai.

### 2.3. Experimental Design and Drug Administration

Five sheep were administered conventional and long acting moxifloxacin formulation at the dose rate of 5.5 and 7.5 mg·kg^−1^ through intravenous route via jugular vein in crossover design, respectively. Washout period of 15 days was kept in between administration of conventional and long acting moxifloxacin. The blood samples were collected using needle (26 G, 0.45 mm × 13 mm) disposable syringe (5 mL). Blood samples (approximately 5 mL) were collected from each treated sheep in heparin containing test tubes with the help of an intravenous catheter (Venflon) fixed into jugular vein at 0 time (before drug administration) and at 0.08 (5 min), 0.25 (15 min), 0.5 (30 min), 1, 2, 4, 8, 12, 24, 36, 48, 60, 72, and 96 h after drug administration. Plasma was separated after centrifugation of blood samples at 1660 revolutions per minute (rpm) for 10 minutes. The plasma samples were transferred to cryovials (3 mL capacity) and stored at −40°C until assayed for long acting moxifloxacin concentration using HPLC procedure.

### 2.4. Moxifloxacin HPLC Assay

Plasma concentrations of moxifloxacin were measured using a modified HPLC method previously reported by Siefert et al. (1999) [6]. The HPLC (AGILENT-1100) system was well equipped with a modal LC-9A (gradient solvent delivery pump), a modal RF-551 fluorescence detector, and a modal SIL-6B automatic sampler and column heater (CTO-6A). Plasma samples were extracted in aliquots by adding 200 *μ*L of plasma to 200 *μ*L of acetonitrile. Plasma proteins were precipitated by shaking in an ultrasonic bath followed by centrifugation for 10 min at 1660 rpm speed. Supernatant was diluted four-fold with 0.067 M disodium hydrogen phosphate buffer with pH 7.5 and transferred to HPLC autosampler vials. 

The HPLC separation was performed with an injection a reserve phase C_18_ (Supelco, 5 *μ*, 4.6 × 150 mm) with using an injection volume of 50 *μ*L. The mobile phase consisted of acetonitrile (20%) and tetrabutyl ammonium hydrogen sulphate solution 10 g/L (80%). Mobile phase was filtered by 0.22 *μ*m filter and degassed by sonicator and then pumped into column at a flow rate of 1.00 mL·min^−1^ at ambient temperature. The fluorescence detection was performed at excitation wavelength of 296 nm excitation and an emission wavelength of 504 nm.

## 3. Results and Discussion

The mean recovery of long acting moxifloxacin from plasma was 85.14% at 25 ng/mL. The sensitivity of long acting moxifloxacin assay was 25 ng·mL^−1^. The assay was sensitive, reproducible, and linearity was observed from 0.025 to 20 *μ*g·mL^−1^. The mean correlation coefficient (*r* ^2^) of long acting moxifloxacin was 0.99997. The lower limit of quantitation (LLOQ) was 25 ng·mL^−1^. 

**Figure 1:**
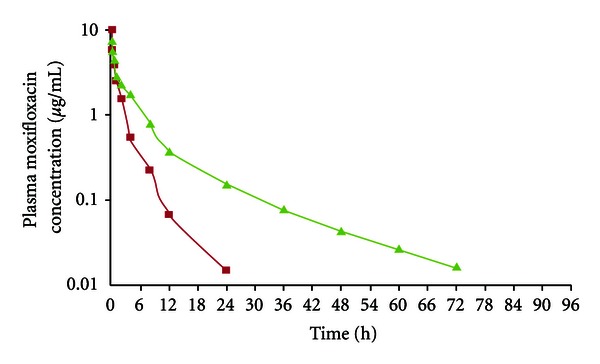
Plasma concentrations of conventional and long-acting moxifloxacin after single-doseintravenous administration in male sheep.

The mean (±SE) plasma concentrations of conventional and long acting moxifloxacin following single-dose intravenous administration are tabulated in Table 1. Mean plasma concentration versus time profile plot of both formulations of moxifloxacin is illustrated in Figure 1. The initial plasma drug concentration of long acting moxifloxacin was 7.212 ± 0.107 *μ*g·mL^−1^ achieved at 0.083 h (5 min). The lowest detectable plasma concentration of long acting moxifloxacin level as 0.016 ± 0.001 *μ*·mL^−1^ was found at 72 h. The therapeutically effective concentration maintained from 0.083 to 72 h. The minimum inhibitory contraction of moxifloxacin is 0.1–0.5 *μ*g·mL^−1^ [7].

Initially, the conventional moxifloxacin plasma level was 9.971 ± 0.901 *μ*g·mL^−1^ recorded at 0.083 h (5 min). The initial concentration of moxifloxacin rapidly declined to 5.596 ± 0.901 *μ*g·mL^−1^ at 0.167 h (15 min). Thereafter, the concentration of the drug gradually diminished and after 24 h it was not detectable in plasma. The plasma concentrations of conventional moxifloxacin at 0.5, 1, 2, 4, 8, 12, and 24 h were 3.887 ± 0.278, 2.496 ± 0.007, 1.529 ± 0.111, 0.544 ± 0.012, 0.224 ± 0.019, 0.067 ± 0.008, and 0.015 ± 0.  *μ*g·mL^−1^, respectively. Thereafter, plasma moxifloxacin level diminished gradually and was not detectable after 24 h of drug administration. 

The initial plasma drug concentration of long acting moxifloxacin was 7.227 ± 0.13 *μ*g·mL^−1^ achieved at 0.083 h (5 min), which rapidly declined to 5.646 ± 0.044 *μ*g·mL^−1^ at 0.166 h (15 min). The plasma concentrations of long acting moxifloxacin at 0.5, 1, 2, 4, 8, 12, 24, 36, 48, 60, and 72 h were 4.283 ± 0.22, 2.853 ± 0.136, 2.230 ± 0.055, 1.712 ± 0.077, 0.772 ± 0.022, 0.368 ± 0.019, 0.150 ± 0.002, 0.076 ± 0.002, 0.043 ± 0.001, 0.026 ± 0.001, and 0.016 ± 0.002 *μ*g·mL^−1^, respectively. Thereafter, plasma long acting moxifloxacin level diminished gradually and was not detectable at 96 h of drug administration. 

While conventional moxifloxacin administration resulted in rapid decline in plasma concentration and the drug was not detectable after 24 hours of administration. This requires frequent administration of drug on daily basis. This would be costly and time consuming. The long acting moxifloxacin administration provided effective therapeutic concentration for up to 72 hours after single-dose administration. This dose is advantageous with respect to cost of treatment and compliance to animal owner. Conversely, while applying long acting moxifloxacin in sheep, initially the levels of drug remained low (7.227 *μ*g·mL^−1^ at 0.083 h) as compared to its counter formulation (conventional) that reached initially to 9.917 *μ*g·mL^−1^ (0.083 h). Since moxifloxacin is a new generation fluoroquinolone antimicrobial drug, with some added quality of initial speedy high level with both types of formulations along with its low MIC level, will definitely make this compound as the drug of choice for the treatment of infectious disorders in sheep.

## Figures and Tables

**Table 1:** Plasma concentrations of conventional long acting moxifloxacin after single-dose intravenous administration in male sheep.

Time after drug administration (h)	Long acting moxifloxacin concentration (*μ*g·mL^−1^)	Conventional moxifloxacin concentration (*μ*g·mL^−1^)
0.083	7.227 ± 0.130	9.917 ± 0.902
0.166	5.646 ± 0.044	5.596 ± 0.092
0.5	4.283 ± 0.220	3.887 ± 0.278
1	2.853 ± 0.136	2.496 ± 0.007
2	2.230 ± 0.055	1.529 ± 0.111
4	1.711 ± 0.077	0.544 ± 0.012
8	0.772 ± 0.022	0.224 ± 0.019
12	0.368 ± 0.019	0.067 ± 0.008
24	0.150 ± 0.002	0.015 ± 0.005
36	0.076 ± 0.002	
48	0.043 ± 0.001	
60	0.026 ± 0.001	
72	0.016 ± 0.002	
96	ND	

ND: not detectable; figures of moxifloxacin concentrations are in mean ± S.E.
